# Machine Learning Based Identification of Depressive Symptoms Among Students in a Chinese University Using Functional Near-Infrared Spectroscopy

**DOI:** 10.31083/AP49235

**Published:** 2025-12-18

**Authors:** Yange Wei, Yuanle Chen, Ning Wang, Huang Zheng, Zhengyun Zhan, Peng Luo, Jinnan Yan, Luhan Yang, Rongxun Liu, Guangjun Ji, Wei Zheng, Yong Meng, Xingliang Xiong

**Affiliations:** ^1^Department of Early Intervention, Mental Health and Artificial Intelligence Research Center, The Second Affiliated Hospital of Xinxiang Medical University, Henan Mental Hospital, 453002 Xinxiang, Henan, China; ^2^Department of Early Intervention, Nanjing Brain Hospital, Nanjing Medical University, 210029 Nanjing, Jiangsu, China; ^3^School of Psychological and Cognitive Sciences, Peking University, 100871 Beijing, China; ^4^Department of Physical Education, Guangdong University of Finance and Economics, 510320 Guangzhou, Guangdong, China; ^5^School of Psychology, Xinxiang Medical University, 453003 Xinxiang, Henan, China; ^6^Department of Psychiatry, The Affiliated Brain Hospital of Guangzhou Medical University, 510631 Guangzhou, Guangdong, China

**Keywords:** depression, NIR spectroscopy, classification, machine learning algorithm, student

## Abstract

**Background:**

Individuals suffer from depression at a high rate on university campuses and current assessment methods primarily rely on subjective questionnaires. Therefore, there is a pressing need to develop objective measures for the automatic detection of depression. This study aimed to investigate the functional near-infrared spectroscopy (fNIRS) changes associated with depression and assess the potential of fNIRS signals in detecting depression among university students.

**Methods:**

A total of 192 participants were recruited for psychological assessment. A 48-channel fNIRS system was employed to measure cerebral blood oxygenation signals during the verbal fluency task (VFT). Two-sample *t*-tests were used to detect group differences. The association between fNIRS data and depression was identified using Pearson correlation analysis. We applied five machine learning classifiers to differentiate depression using fNIRS signals. Model performance was evaluated using receiver operating characteristic (ROC) curves, area under the curve (AUC), precision, accuracy, recall, and F1 score. A ten-fold cross-validation incorporating the recursive feature elimination algorithm was utilized.

**Results:**

Significant hemodynamic alterations were observed in the depression group at channels 4, 16, 21, 26, 32, 43, 44, and 47, in comparison with the control group. The bilateral medial prefrontal cortices (MPFC), left dorsolateral prefrontal cortex, and left temporal lobe, represented by channels 4, 16, 43, and 44, were associated with depression. Among the five machine learning algorithms, K-Nearest-Neighbors (KNN) exhibited superior classification performance (AUC = 66.51%). The left MPFC was the most significant contributor to the classification efficacy of the KNN model.

**Conclusion:**

fNIRS-VFT may serve as an objective tool for evaluating depressive symptoms in university students. The findings underscore the central role of the left MPFC in the neural mechanisms underlying depression. This work developed an fNIRS-based identification system for depression in university students.

## Main Points

1. Chinese university students with depressive symptoms exhibited hemodynamic changes in bilateral medial prefrontal cortices (MPFC), left dorsolateral prefrontal cortex, and left temporal lobe.

2. Among the five machine learning algorithms, the K-Nearest Neighbors (KNN) model demonstrated optimal performance in differentiating depressed and non-depressed university students.

3. The left MPFC contributed most to the KNN model’s classification accuracy, suggesting its crucial role in depression.

## 1. Introduction

The prevalence of depression is increasing and constitutes a significant mental health issue on Chinese university campuses. Factors such as separation from parents, inadequate adaptation to new environments, academic stress, and career planning challenges contribute to this phenomenon, with approximately 15% to 35% of Chinese university students report experiencing depression [1]. Early identification is essential to mitigate the adverse effects of depressive symptoms on students’ academic and occupational performance [2]. Currently, the detection of depressive symptoms predominantly relies on self-reported questionnaires, including the Patient Health Questionnaire-9 (PHQ-9), the Hamilton Depression Rating Scale (HAMD), and Beck’s Depression Inventory (BDI). These tools may introduce subjective bias into the assessment results, and their flexibility and accuracy are relatively limited [3]. Consequently, there is an urgent need for objective, quantitative measures that can accurately identify depressive symptoms in Chinese university students and elucidate their underlying neural mechanisms.

Researchers are increasingly developing technological tools to address the challenges described. Functional near-infrared spectroscopy (fNIRS), a non-invasive brain imaging technique based on neurovascular coupling, is widely used to understand the neurobiology of depression [4]. Compared with electroencephalography and functional magnetic resonance imaging, fNIRS has many advantages, such as relatively low cost, safety, portability, high temporal resolution, and insensitivity to motion artifacts [5]. fNIRS can utilize the tight coupling between oxygenation levels associated with neural activity and localized cerebral blood flow to monitor hemodynamic changes [6]. Therefore, it has the potential to provide mechanistic insights into the neurobiological alterations associated with depression [4]. The verbal fluency task (VFT) is often used with fNIRS to evaluate an individual’s ability to convey information verbally within a defined timeframe or category, serving as an indicator of linguistic competence and cognitive flexibility [2, 7]. Hence, the fNIRS-VFT paradigm offers a promising approach to explore the neural mechanisms of depression [8]. Previous study has reported significant hemodynamic changes in depression utilizing the fNIRS-VFT paradigm, further supporting the link between fNIRS signals and depression [9]. Given the distinct linguistic characteristics and retrieval strategies inherent in English and Chinese [2, 10, 11], the findings derived from the fNIRS-English VFT paradigm are not directly transferable to the Chinese population. Therefore, we employed the fNIRS-Chinese VFT paradigm as an objective measure to investigate the neural activity of brain regions in Chinese university students.

Rapid and accurate methodologies are required for large-scale depression screening among the Chinese university student population. With the development of artificial intelligence, machine learning algorithms offer a powerful approach to automatically learn from existing data, establish functional relationships, identify latent patterns, and make predictions [12]. Previous studies have utilized fNIRS to develop machine learning models that discriminate depression by employing various feature combinations during the VFT [13, 14]. A recent study employed fNIRS and support vector machine (SVM) to classify mild and severe depression in a cohort of 140 subjects [14]. Yi *et al*. [13] used fNIRS signals from 25 depressed individuals and 30 healthy controls during a resting state to develop an SVM model for the automatic classification of depression. However, fewer studies have utilized fNIRS in the context of the Chinese VFT to construct machine learning models for identifying depressive symptoms in Chinese university students.

This study aimed to develop an fNIRS-based system for the identification of depressive symptoms among Chinese university students by employing five distinct machine learning algorithms. The primary objective was to identify specific hemodynamic alterations in Chinese university students exhibiting depressive symptoms. Furthermore, the study aimed to investigate the correlation between regional hemodynamic changes and the severity of depressive symptoms. Lastly, we aimed to evaluate the efficacy of machine learning models in differentiating between individuals with and without depressive symptoms based on fNIRS data.

## 2. Material and Methods

### 2.1 Participants and Psychological Assessment

This cross-sectional study was implemented via an online survey conducted from March to May, 2024. Participants were recruited through poster advertisements at Xinxiang Medical University, located in Xinxiang, Henan, China. The research team contacted those who were willing to participate in the study and provided a detailed explanation of the procedures. Inclusion criteria for participants with depressive symptoms were as follows: (i) aged between 18 and 30 years; (ii) PHQ-9 score ≥5 [15, 16]; (iii) able to read and understand Chinese; and (iv) no prior treatment with antidepressant medication, neurostimulation therapy, or evidence-based psychotherapy. Exclusion criteria included: (i) a history of mental disorders or substance abuse; (ii) a history of neurological disorders; and (iii) a primary language other than Chinese.

**Fig. 1.**
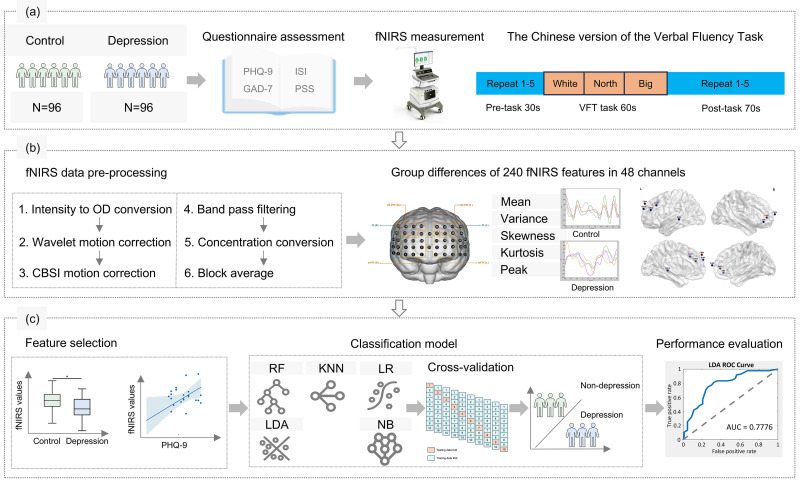
**Flowchart outlining the study workflow**. (a) The functional near-infrared spectroscopy (fNIRS) dataset was obtained from 96 controls and 96 depressions from Chinese university students. (b) After fNIRS data pre-processing, a total of 240 fNIRS features for each subject were obtained. (c) Followed by feature selection of fNIRS signals, machine learning analyses utilized five classifiers with 10-fold cross-validation. Abbreviations: PHQ-9, Patient Health Questionnaire-9; GAD-7, Generalized Anxiety Disorder-7; PSS, Perceived Stress Scale; ISI, Insomnia Severity Index; OD, optical density; CBSI, correlation-based signal improvement; KNN, K-Nearest-Neighbors; RF, Random Forest; LDA, Linear Discriminant Analysis; LR, Logistic Regression; NB, Naive Bayes; ROC, receiver operating characteristic; AUC, area under the curve; VFT, verbal fluency task.

All psychological questionnaires were completed on the WeChat-based official account platform. The severity of depression was assessed using the PHQ-9 [17, 18, 19, 20], anxiety by the Generalized Anxiety Disorder-7 (GAD-7), sleep by the Insomnia Severity Index (ISI), and stress by the Perceived Stress Scale (PSS). The study design is depicted in Fig. 1.

### 2.2 fNIRS Measurement

**Fig. 2.**
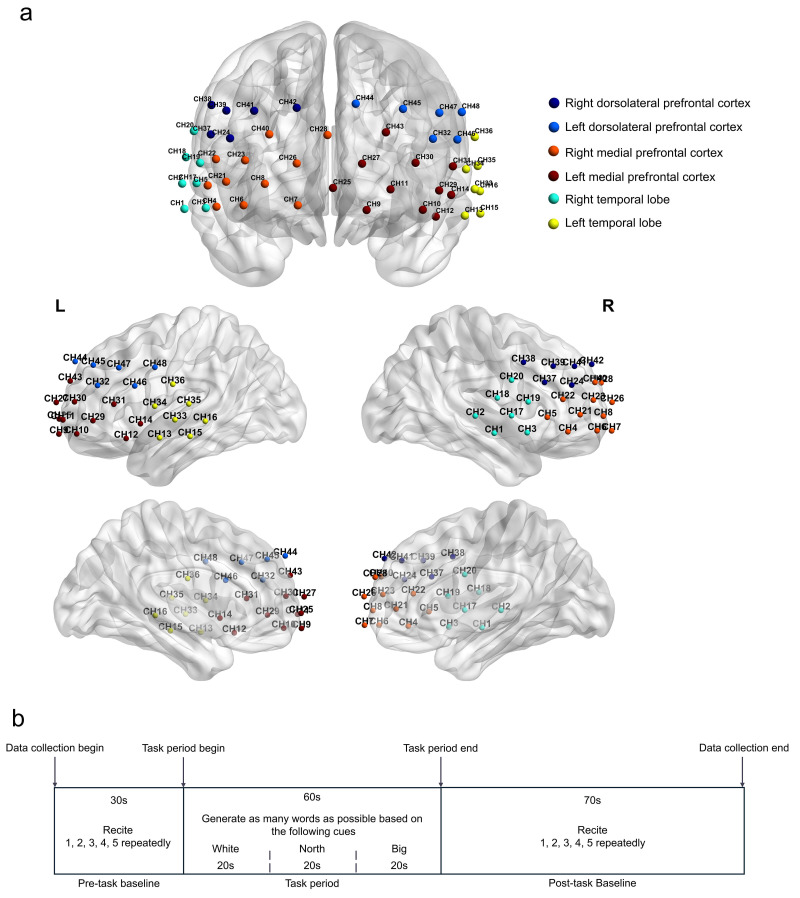
**Channel information and the verbal fluency task protocol of this study**. (a) Schematic of fNIRS channel and optode arrangement. (b) The verbal fluency task process. Each trial comprised a 30-second pre-task rest period, a 60-second task period (divided into three 20-second blocks), and a 70-second post-task rest period.

The fNIRS experiment was conducted in a controlled environment characterized by darkness and the absence of noise. Participants were instructed to minimize head movements and maintain emotional stability throughout the experiment. A 48-channel fNIRS system (NirScan, Danyang Huichuang Medical Equipment Co., Ltd., Danyang, Jiangsu, China) was employed in this study. The arrangement of channels and probes is displayed in Fig. 2a. The Chinese version of the VFT was employed to assess verbal fluency, working memory, verbal recall, attention, and retrieval [21]. The VFT task consisted of three distinct phases: a 30-second pre-task baseline period, a 60-second VFT task period, and a 70-second post-task period [22]. The baseline was defined as a period when participants did not perform a VFT task. During the 30-second pre-task baseline phase, participants were instructed to repeatedly count from one to five until the task period began. During the 60-second VFT task period, participants were instructed to generate as many phrases as possible using simple words such as “white”, “north”, and “big”. After completing the phrase constructions, participants were instructed to resume counting from one to five repeatedly for the duration of the 70-second post-task period. The overall structure of the VFT task is depicted in Fig. 2b. Oxyhemoglobin, deoxyhemoglobin, and total hemoglobin were measured to quantify hemodynamic changes. Fifteen light source probes and sixteen light detector probes were positioned on the bilateral frontotemporal cortex, maintaining a 3.0 cm distance between each light source and detector probe. In accordance with the 10/20 electrode placement system, the center of the middle probe was aligned with frontopolar midline (FPz), while the lower edge of the probe array extended from Fp1 to Fp2.

### 2.3 fNIRS Data Processing

Raw fNIRS data were preprocessed using the Homer 3 toolkit in MATLAB R2023b (The MathWorks, Inc., Natick, MA, USA). The preprocessing protocol encompassed the following steps: (i) converting light intensity values to optical density values; (ii) identification and removal of motion artifacts; (iii) application of the correlation-based signal improvement motion correction method to mitigate the effects of movement on the signal; (iv) filtering of the signal to eliminate physiological and instrumental noise; (iv) filtering of the signal to eliminate physiological and instrumental noise; (v) concentration conversion and perform block average process based on the marker with the range set to 30 seconds before the marker and 60 seconds after the marker; and (vi) calculation of five statistical measures of fNIRS, including mean, variance, skewness, kurtosis, and peak value, derived from temporal analysis of changes in the oxyhemoglobin signal across all 48 channels. This yielded a total of 240 independent features for each subject. The 3D localization process and Montreal Neurological Institute (MNI) coordinates for each optode and channel were provided by the manufacturer of the fNIRS device. Spatial registration of the channels was conducted using the MATLAB toolkit Statistical Parametric Mapping (https://www.nitrc.org/projects/nirs_spm/) for Near-infrared Spectroscopy v4 (NIRS-SPM) [23] to estimate anatomical labels. The Brodmann Area anatomical template was applied to determine the percentage of overlap with the corresponding brain regions. MNI spatial information was visualized using the MATLAB toolkit BrainNet Viewer 1.63 (http://www.nitrc.org/projects/bnv/) with the International Consortium for Brain Mapping 152-subject average head model (ICBM-152) [24]. Spatial information, MNI coordinates, and the brain region of each of the 48 channels are shown in Table 1 and Fig. 2a.

**Table 1.** **MNI coordinates, corresponding brain region, and percentage of overlap of each channel**.

Channel number	MNI			Brodmann Area-anatomical label	Percentage of overlap
	X	Y	Z		
1	70	–11	–11	21 - Middle temporal gyrus	0.972
				22 - Superior temporal gyrus	0.028
2	71	–24	2	21 - Middle temporal gyrus	0.569
				22 - Superior temporal gyrus	0.431
3	60	12	–10	21 - Middle temporal gyrus	0.212
				38 - Temporopolar area	0.770
				48 - Retrosubicular area	0.018
4	55	41	–10	38 - Temporopolar area	0.025
				45 - Pars triangularis Broca’s area	0.209
				46 - Dorsolateral prefrontal cortex	0.323
				47 - Inferior prefrontal gyrus	0.443
5	59	26	1	38 - Temporopolar area	0.320
				44 - Pars opercularis, part of Broca’s area	0.010
				45 - Pars triangularis Broca’s area	0.521
				48 - Retrosubicular area	0.149
6	42	62	–9	10 - Frontopolar area	0.284
				11 - Orbitofrontal area	0.114
				46 - Dorsolateral prefrontal cortex	0.271
				47 - Inferior prefrontal gyrus	0.331
7	16	72	–9	10 - Frontopolar area	0.139
				11 - Orbitofrontal area	0.861
8	32	66	1	10 - Frontopolar area	0.543
				11 - Orbitofrontal area	0.457
9	–18	70	–11	10 - Frontopolar area	0.011
				11 - Orbitofrontal area	0.989
10	–45	58	–11	10 - Frontopolar area	0.101
				46 - Dorsolateral prefrontal cortex	0.535
				47 - Inferior prefrontal gyrus	0.364
11	–29	68	–1	10 - Frontopolar area	0.447
				11 - Orbitofrontal area	0.553
12	–51	23	–14	38 - Temporopolar area	0.987
				47 - Inferior prefrontal gyrus	0.013
13	–65	–1	–14	21 - Middle temporal gyrus	0.980
				38 - Temporopolar area	0.020
14	–58	13	–4	21 - Middle temporal gyrus	0.028
				38 - Temporopolar area	0.638
				48 - Retrosubicular area	0.334
15	–72	–22	–13	20 - Inferior temporal gyrus	0.146
				21 - Middle temporal gyrus	0.854
16	–72	–33	–2	20 - Inferior temporal gyrus	0.096
				21 - Middle temporal gyrus	0.667
				22 - Superior temporal gyrus	0.237
17	64	1	2	6 - Pre-motor and supplementary motor cortex	0.028
				21 - Middle temporal gyrus	0.224
				22 - Superior temporal gyrus	0.070
				38 - Temporopolar area	0.070
				48 - Retrosubicular area	0.608
18	69	–9	14	22 - Superior temporal gyrus	0.539
				43 - Subcentral area	0.373
				48 - Retrosubicular area	0.088
19	63	13	12	6 - Pre-motor and supplementary motor cortex	0.455
				44 - Pars opercularis, part of Broca’s area	0.296
				45 - Pars triangularis Broca’s area	0.022
				48 - Retrosubicular area	0.226
20	66	1	27	4 - Primary motor cortex	0.068
				6 - Pre-motor and supplementary motor cortex	0.357
				43 - Subcentral area	0.575
21	50	50	2	45 - Pars triangularis Broca’s area	0.131
				46 - Dorsolateral prefrontal cortex	0.869
22	55	37	13	45 - Pars triangularis Broca’s area	0.983
				46 - Dorsolateral prefrontal cortex	0.017
23	41	59	13	10 - Frontopolar area	0.444
				46 - Dorsolateral prefrontal cortex	0.556
24	48	44	23	45 - Pars triangularis Broca’s area	0.771
				46 - Dorsolateral prefrontal cortex	0.229
25	–1	70	–1	10 - Frontopolar area	0.865
				11 - Orbitofrontal area	0.135
26	16	72	11	10 - Frontopolar area	1.000
27	–15	72	11	10 - Frontopolar area	1.000
28	1	64	25	10 - Frontopolar area	1.000
29	–53	46	–2	45 - Pars triangularis Broca’s area	0.349
				46 - Dorsolateral prefrontal cortex	0.647
				47 - Inferior prefrontal gyrus	0.004
30	–41	59	12	10 - Frontopolar area	0.366
				46 - Dorsolateral prefrontal cortex	0.634
31	–59	32	10	45 - Pars triangularis Broca’s area	1.000
32	–50	43	23	45 - Pars triangularis Broca’s area	0.912
				46 - Dorsolateral prefrontal cortex	0.088
33	–70	–12	–1	21 - Middle temporal gyrus	0.547
				22 - Superior temporal gyrus	0.419
				48 - Retrosubicular area	0.034
34	–65	3	8	6 - Pre-motor and supplementary motor cortex	0.277
				22 - Superior temporal gyrus	0.039
				38 - Temporopolar area	0.003
				43 - Subcentral area	0.072
				48 - Retrosubicular area	0.609
35	–71	–21	10	21 - Middle temporal gyrus	0.193
				22 - Superior temporal gyrus	0.807
36	–70	–10	24	2 - Primary somatosensory cortex	0.063
				22 - Superior temporal gyrus	0.094
				43 - Subcentral area	0.724
				48 - Retrosubicular area	0.119
37	58	24	25	44 - Pars opercularis, part of Broca’s area	0.434
				45 - Pars triangularis Broca’s area	0.566
38	57	10	39	4 - Primary motor cortex	0.004
				6 - Pre-motor and supplementary motor cortex	0.597
				9 - Dorsolateral prefrontal cortex	0.078
				44 - Pars opercularis, part of Broca’s area	0.322
39	50	31	37	9 - Dorsolateral prefrontal cortex	0.099
				44 - Pars opercularis, part of Broca’s area	0.332
				45 - Pars triangularis Broca’s area	0.486
				46 - Dorsolateral prefrontal cortex	0.083
40	29	60	25	9 - Dorsolateral prefrontal cortex	0.016
				10 - Frontopolar area	0.433
				46 - Dorsolateral prefrontal cortex	0.551
41	37	46	37	9 - Dorsolateral prefrontal cortex	0.453
				45 - Pars triangularis Broca’s area	0.014
				46 - Dorsolateral prefrontal cortex	0.533
42	16	58	38	9 - Dorsolateral prefrontal cortex	0.851
				10 - Frontopolar area	0.129
				46 - Dorsolateral prefrontal cortex	0.020
43	–27	62	26	9 - Dorsolateral prefrontal cortex	0.004
				10 - Frontopolar area	0.416
				46 - Dorsolateral prefrontal cortex	0.580
44	–12	59	40	9 - Dorsolateral prefrontal cortex	0.885
				10 - Frontopolar area	0.115
45	–35	46	38	9 - Dorsolateral prefrontal cortex	0.456
				45 - Pars triangularis Broca’s area	0.005
				46 - Dorsolateral prefrontal cortex	0.539
46	–61	17	23	6 - Pre-motor and supplementary motor cortex	0.248
				44 - Pars opercularis, part of Broca’s area	0.614
				45 - Pars triangularis Broca’s area	0.138
47	–53	28	36	44 - Pars opercularis, part of Broca’s area	0.548
				45 - Pars triangularis Broca’s area	0.452
48	–63	3	36	3 - Primary somatosensory cortex	0.019
				4 - Primary motor cortex	0.177
				6 - Pre-motor and supplementary motor cortex	0.577
				43 - Subcentral area	0.226

Abbreviations: MNI, Montreal Neurological Institute.

### 2.4 Statistical Analysis

In this study, a total of 192 participants (96 per group) were recruited to ensure adequate statistical power and reliability of the results. Statistical analysis was performed using MATLAB R2017a (The MathWorks, Inc.). Data distribution was examined for normality using the Kolmogorov-Smirnov test. Demographic, scales, and VFT performance data were analyzed by independent *t*-test, Mann–Whitney U test, and chi-squared test. Descriptive statistics for normally distributed variables were reported as mean ± standard deviation (SD), whereas non-normally distributed variables were reported as median and interquartile range (IQR). We measured significant changes of 240 fNIRS features in university students with depression compared with controls. Then, two-tailed paired *t*-tests and Pearson correlation analyses were conducted to examine the relationship between significantly altered fNIRS features and PHQ-9 scores. *p* < 0.05 was considered to be statistically significant.

### 2.5 Machine Learning Algorithms for Classification

Machine learning analysis involved feature selection and model construction utilizing the K-Nearest-Neighbors (KNN), Random Forest (RF), Linear Discriminant Analysis (LDA), Logistic Regression (LR), and Naive Bayes (NB) algorithms, along with k-fold cross-validation. For feature selection, we hypothesized that fNIRS features with significant alterations and associations with depression might have better discriminative ability. Consequently, we used two-tailed student’s *t*-tests and Pearson correlation analyses to identify statistically significant fNIRS features as input. The parameters of the five machine learning algorithms were as follows. In KNN, number of Neighbors: 9, distance metric: ‘cosine’, number of folds: 4; In RF, number of trees: 130, minimum leaf size: 17, number of folds: 2; In LDA, gamma: 0.0689, number of folds: 10; In LR, regularization parameter lambda: 0.2918, number of folds: 10; in NB, distribution type: ‘kernel’, kernel type: ‘normal’, kernel width: 0.3, number of folds: 8, respectively. In accordance with previous machine learning research [25], we employed ten-fold cross-validation to validate model results and avoid overfitting. Model performance was evaluated using the receiver operating characteristic (ROC) curve, from which the area under the curve (AUC) was calculated, as well as precision, accuracy, recall, and F1 score. Five machine learning algorithms were evaluated, and the algorithm exhibiting the highest AUC value was selected as the optimal model for identifying depression among university students. Finally, recursive feature elimination was employed to rank the features according to their importance. The models were programmed in MATLAB R2017a (The MathWorks, Inc.).

## 3. Results

### 3.1 Demographic Characteristics

According to the sample size calculation, a minimum of 58 participants was required to achieve adequate statistical power. To enhance statistical power and ensure the reliability of the results, the study included 96 participants with depressive symptoms and 96 controls. The depression group demonstrated significantly higher scores on the PHQ-9, GAD-7, ISI, and PSS scales compared with the control group (*p* < 0.05). No significant differences were observed between the groups in terms of age, gender, or education level (*p* > 0.05). For VFT performance, the depression group tended to generate fewer words than the control group, although these differences were not statistically significant (*p* > 0.05). See Table 2.

**Table 2.** **Demographic characteristics of controls and depressive symptoms among university students in China**.

Characteristic		Control N = 96	Depression N = 96	Statistics	*p* values
Age, years		21 (3)	20.5 (2)	4176	0.251^a^
Gender (Female/Male)		72/24	69/27	0.240	0.624^c^
Education level (Year)		15 (3)	15 (3)	4295	0.357^a^
PHQ-9 score		2 (3)	8 (4)	9216	<0.001*^a^
GAD-7 score		0 (1)	5 (5)	8500	<0.001*^a^
PSS score		15.42 ± 6.09	26.06 ± 8.55	–9.935	<0.001*^b^
ISI score		2 (3)	7.5 (6)	8041	<0.001*^a^
VFT performance					
	White	4 (3)	4 (2)	3820	0.164^a^
	North	4 (2)	4 (2)	3956	0.307^a^
	Big	4 (3)	4 (3)	4221	0.777^a^
	Total	13 (6)	12 (6)	4093	0.253^a^

Notes: Continuous data are presented as mean (SD) and categorical data as n (%). ^a^ Mann–Whitney U test, ^b^ Independent *t*-test, ^c^ Chi-squared test. Normally distributed data are expressed as mean ± standard deviation (SD). Non-normally distributed variables are reported as median and interquartile range (IQR). *p*-values for between-group comparisons were determined based on the results of normality assessment using the Kolmogorov–Smirnov test. *Significance level was set at *p* < 0.05. Abbreviations: PHQ-9, Patient Health Questionnaire-9; GAD-7, Generalized Anxiety Disorder-7; PSS, Perceived Stress Scale; ISI, Insomnia Severity Index.

### 3.2 Significant fNIRS Changes in Depression

**Fig. 3.**
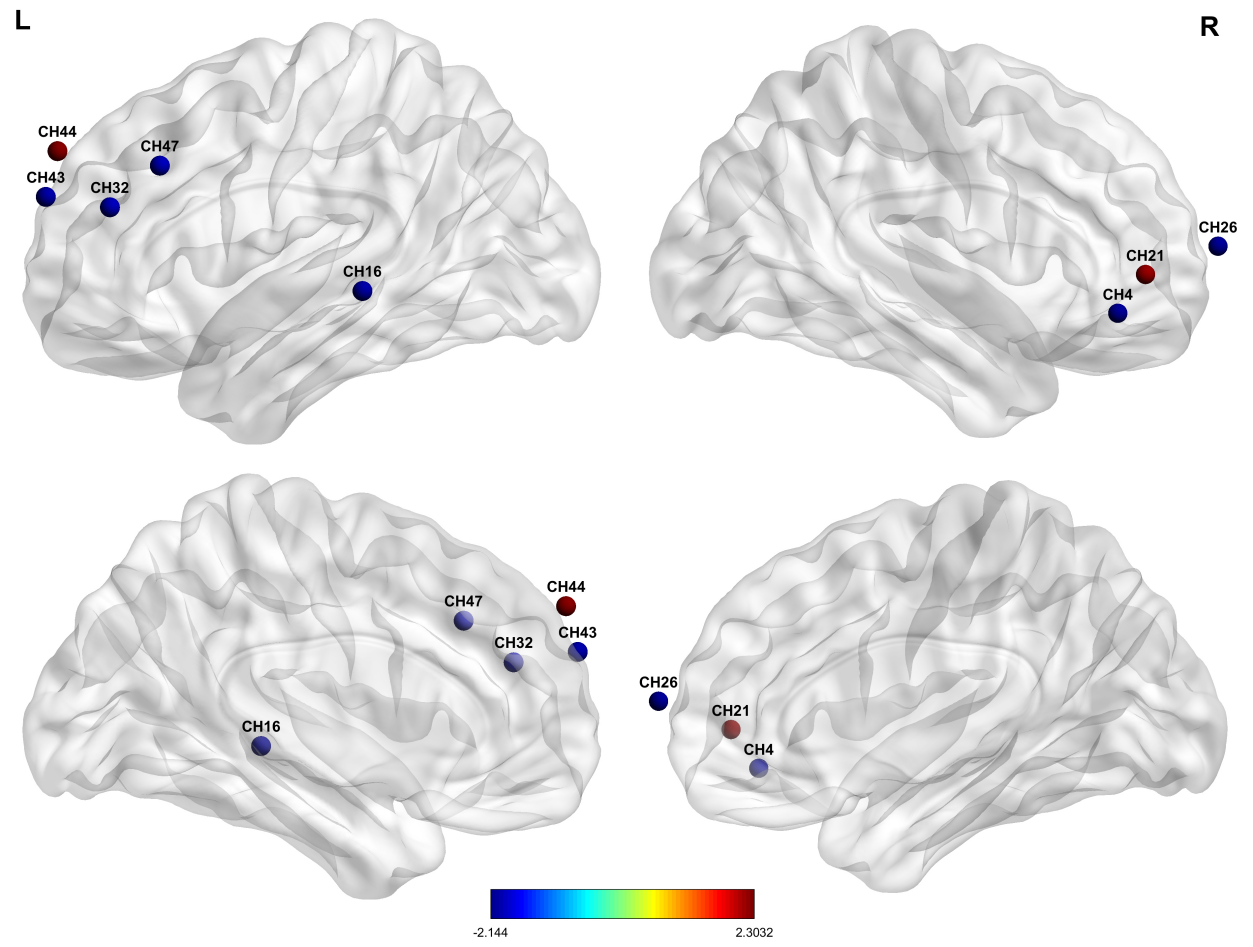
**Cerebral hemodynamic differences between individuals with depressive symptoms and the control group**. Red nodes indicate relatively higher oxygenated hemoglobin levels; blue denotes relatively lower oxygenated hemoglobin levels in individuals with depressive symptoms, compared with controls. The significance was set at *p* < 0.05. The color bar indicates the t-value of the brain area. The labels “L” and “R” indicate the left and right hemispheres of the brain, respectively.

**Fig. 4.**
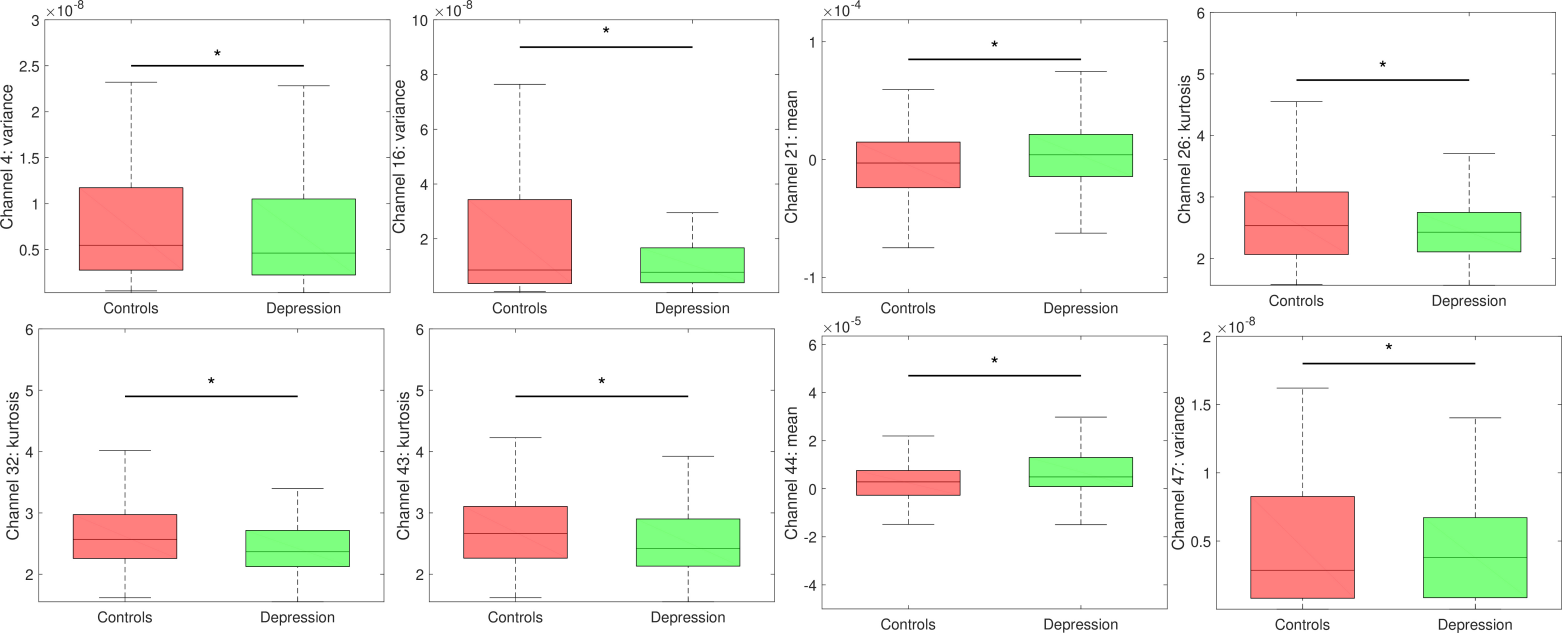
**Significant hemodynamic alterations in Chinese university students with depressive symptoms compared with controls**. Box charts depicting the mean hemodynamic values of the control (red) and depression groups (green). Asterisks denote statistical significance at *p* < 0.05.

Eight fNIRS features exhibited significant differences between the control and depression group (*p* < 0.05). Compared with controls, individuals with depression demonstrated significantly lower variance in channels 4, 47, and 16, as well as kurtosis in channels 26, 32, and 43, while showing higher mean values in channels 21 and 44. Collectively, the bilateral medial prefrontal cortices (MPFC), left dorsolateral prefrontal cortex (DLPFC), and left temporal lobe (TL), represented by channels 4, 16, 21, 26, 32, 43, 44, and 47, showed significant hemodynamic changes in depression. See Figs. 3,4.

### 3.3 Correlation Between Significant fNIRS Changes and Depression

PHQ-9 scores were negatively associated with variance values on channel 4 (R = –0.167, *p *= 0.021) and channel 16 (R = –0.170, *p *= 0.019) and kurtosis values on channel 43 (R = –0.178, *p *= 0.014), and positively associated with mean values on channel 44 (R = 0.177, *p *= 0.014). Together, bilateral MPFC (channels 4 and 43), left DLPFC (channel 44), and TL (channel 16) were significantly associated with the severity of depressive symptoms. See Table 3.

**Table 3.** **Correlation between functional near-infrared spectroscopy values and PHQ-9 scores**.

Brain area	Brodmann	Channel	MNI coordinates	Variable	R value	R^2^ value	*p* value
Right medial prefrontal cortex	BA47	4	55, 41, –10	Variance	–0.167	0.028	0.021
Left temporal lobe	BA21	16	–72, –33, –2	Variance	–0.170	0.029	0.019
Left medial prefrontal cortex	BA10	43	–27, 62, 26	Kurtosis	–0.178	0.032	0.014
Left dorsolateral prefrontal cortex	BA9	44	–12, 59, 40	Mean	0.177	0.031	0.014

Abbreviations: BA, brodmann area.

### 3.4 Classification Results

**Fig. 5.**
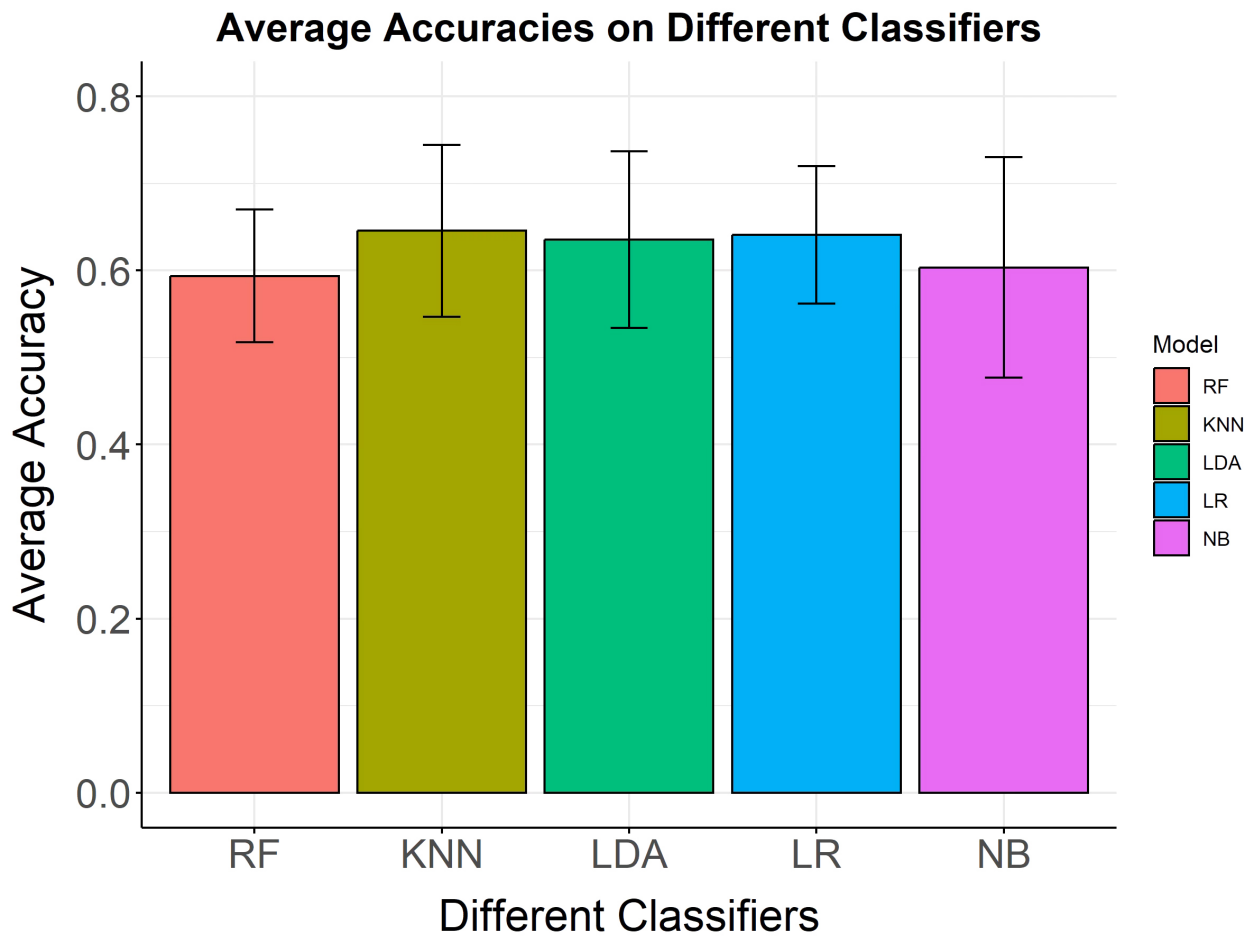
**Average classification accuracies and standard deviations on five classifiers**. Average classification accuracies and standard deviations on five classifiers. The y-axis shows the mean accuracy, while the x-axis lists the classifiers.

**Fig. 6.**
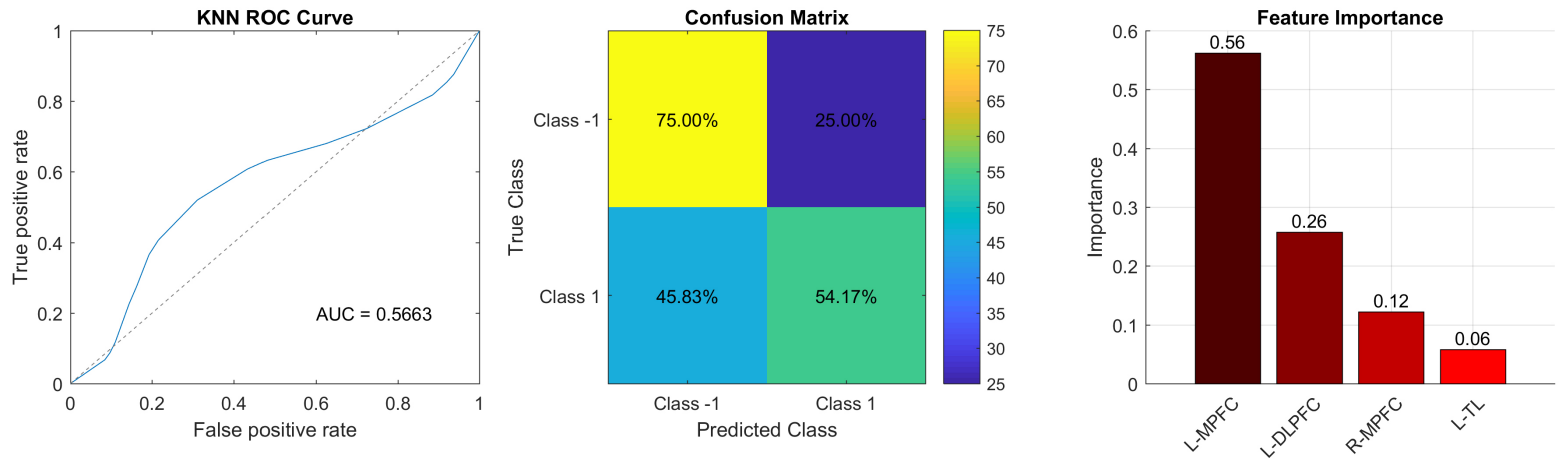
**Classification performance of the optimal model**. ROC curve (left), confusion matrix (middle), and feature importance (right). Abbreviations: MPFC, medial prefrontal cortex; DLPFC, dorsolateral prefrontal cortex; TL, temporal lobe.

**Fig. 7.**
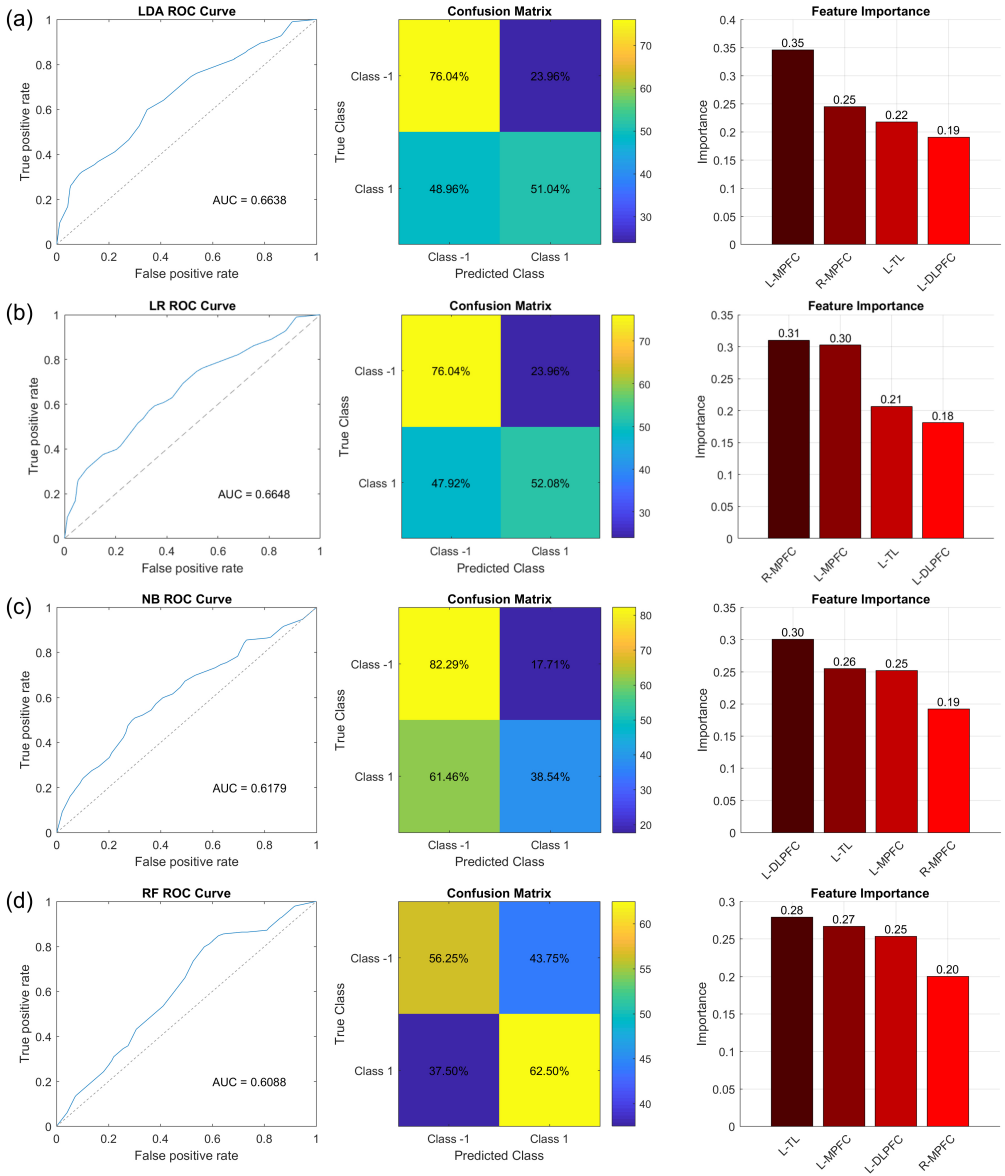
**Classification performance of LDA, LR, NB, and RF**. ROC curve (left), confusion matrix (middle), and feature importance (right) of LDA (a), LR (b), NB (c), and RF (d).

Based on the above results, four features-variance values in channels 4 and 16, kurtosis values in channel 43, and mean values in channel 44 were utilized as inputs for the machine learning algorithm. The analysis of the ROC curves and confusion matrices indicates that the average AUC of five machine learning algorithms consistently exceeds 60% for all classifiers (Fig. 5). Notably, the KNN model exhibited superior classification performance, with an accuracy of 65.63%, an AUC of 66.51%, precision of 66.97%, recall of 61.46%, and an F1 score of 63.73%, outperforming the RF, LDA, LR, and NB. In terms of feature importance, kurtosis in channel 43 (left MPFC) was the most significant contributor to the optimal KNN model’s classification efficacy. This fNIRS feature was located in the left MPFC. The ROC curves, confusion matrix, and feature importance are shown in Table 4, and Figs. 6,7.

**Table 4.** **Classification results**.

Classifier	Accuracy	AUC	Precision	Recall	F1
KNN	65.63%	66.51%	66.97%	61.46%	63.73%
RF	63.02%	64.61%	63.73%	61.46%	62.42%
LDA	63.55%	66.38%	70.33%	51.56%	56.83%
LR	64.08%	66.48%	70.99%	52.67%	57.33%
NB	60.94%	63.59%	65.79%	47.92%	54.75%

## 4. Discussion

This study developed an fNIRS-based system to identify depressive symptoms among Chinese university students by utilizing five machine learning algorithms. There were three main findings of our study. First, individuals with depression exhibit significant hemodynamic changes in the bilateral MPFC, left DLPFC, and TL compared with controls, suggesting that these regions are involved in cognitive processing during a VFT. Second, fNIRS signals in these brain regions were also significantly associated with depression, highlighting their critical role in the neural mechanisms underlying depression. Third, these distinct fNIRS alterations were utilized to develop classification models for identifying depressive symptoms among university students. Among the five machine learning methods, the KNN algorithm exhibited the highest performance, with a mean AUC of 66.51% and an accuracy of 65.63%. Notably, the left MPFC contributed the most to the KNN model’s classification accuracy, highlighting its crucial role in depression. These findings suggest that the integration of fNIRS with machine learning classifiers could serve as an objective and precise tool to complement traditional diagnostic methods.

In individuals with depression, significant hemodynamic changes were observed in the bilateral MPFC, left DLPFC, and left TL, as represented by channels 4, 16, 21, 26, 32, 43, 44, and 47, which were also correlated with PHQ-9 scores. These findings regarding hemodynamic alterations are consistent with previous research. Lim and Park [26] investigated the hemodynamic changes in individuals with subclinical depression, identifying significant differences in the prefrontal cortex. Sun *et al*. [27] observed decreased activation in the DLPFC among individuals with depressive symptoms. Similarly, Fan *et al*. [9] reported diminished activation in the prefrontal cortex, particularly within the DLPFC in depressed individuals. Yang *et al*. [28] also demonstrated reduced activity in the left TL and DLPFC among individuals with depression. A recent study involving 72 depressed university students identified a significant correlation between the left DLPFC and depression [29]. The MPFC, DLPFC, and TL have been implicated in cognitive, emotional, and behavioral regulation [30, 31, 32]. The VFT task activated the prefrontal cortex, including the MPFC and DLPFC, which are primarily responsible for emotional regulation and cognitive control, language comprehension, and memory retrieval [33, 34]. Depressed individuals may be unable to effectively mobilize the full functionality of the MPFC, DLPFC, and TL, leading to correspondingly flattened brain responses and low variability during the VFT task. Chinese university students experiencing depression demonstrated significant alterations in the MPFC, DLPFC, and TL, potentially linked to impaired emotional regulation and cognitive control [30, 31, 32]. Consequently, we speculate that dysfunction in the MPFC, DLPFC, and TL contributed to the observed symptoms in individuals with depression, such as flat affect, low mood, delayed decision-making, weakened executive functions, and challenges in language generation and semantic retrieval. Frontotemporal hemodynamic changes detected by fNIRS during VFT may serve as an objective measure for assessing depression among Chinese university students. Previous studies have reported similar findings. In one study involving 72 depressed university students and 67 healthy controls using fNIRS during a VFT, the depression group exhibited lower oxyhemoglobin levels in the right DLPFC and Broca’s area, along with reduced frontotemporal connectivity. Importantly, these alterations were correlated with the severity of depression [35]. Park *et al*. [36] identified decreased activation in the right frontopolar cortex and MPFC in the depression group. In addition, a recent study conducted by Kang *et al*. [37], involving 204 older adults with depression revealed diminished prefrontal hemodynamic responses during the Stroop test. However, no significant differences were observed in performance on the digit span backward task or the VFT. Collectively, these findings suggested that frontal hemodynamic changes detected by fNIRS during a VFT have the potential to be an objective measure for assessing depressive symptoms among Chinese university students.

We employed five distinct machine learning algorithms to capitalize on their unique strengths and facilitate a comparative analysis using a consistent dataset. Each algorithm exhibits specific characteristics that may yield different performance. This diverse selection of classifiers ensures a balanced evaluation encompassing linear, nonlinear, probabilistic, and instance-based learning methodologies, thereby enabling a comprehensive assessment of classification performance and model generalizability in the context of depression-related data analysis. The KNN model demonstrated the highest classification accuracy. Owing to its low computational demands, straightforward implementation, and robust classification performance, KNN is a prevalent and effective classification technique in the fields of data mining and statistics [38]. However, the classification accuracy achieved by the KNN model did not attain an ideal level. Several factors may have contributed to this outcome. First, the feature extraction and selection processes from the fNIRS data may not have fully captured all critical neural activity patterns associated with depression. Despite utilizing a grid search strategy to optimize model parameters, parameter selection remains inadequate for achieving optimal results. Second, the hemodynamic changes reflected in the fNIRS data may have been influenced by biological variables, such as individual differences, environmental factors, or experimental conditions, all of which can impact the model’s accuracy. Third, this study primarily concentrated on binary classification (depression versus control) without stratifying participants according to depression severity scores. Indeed, we observed fewer differences between university students with depressive symptoms and controls than between patients with clinical depression and controls. This may be due to the mild depressive symptoms exhibited by participants, as defined by a PHQ-9 score of ≥5 for Chinese university students. Future research will incorporate a grading system for depression severity, potentially utilizing the PHQ-9 or other standardized clinical assessments, to improve classification accuracy and explore whether varying severity levels manifest distinct neurobiological or behavioral characteristics. Moreover, deep learning models, particularly Convolutional Neural Networks (CNNs), are capable of effectively capturing highly non-linear relationships in data through their multi-layer neural structures [39]. These models are adept at identifying and extracting significant features from data automatically [40], thereby reducing the dependency on manual feature engineering when processing complex data types such as images, audio, and time-series data [41]. To improve the predictive performance of models, we will integrate CNNs with attention mechanisms to more effectively capture spatial and temporal patterns in fNIRS signals.

The left MPFC contributed the most to the optimal KNN model’s classification accuracy, highlighting the crucial role of the left MPFC regions in the neural mechanisms of depression [42]. The MPFC is involved not only in emotional regulation but also in cognitive control processes including decision-making and impulse control [30]. Its functional role is closely linked to the brain’s functional lateralization, with the left hemisphere typically associated with positive emotion processing. Whereas the right hemisphere is more engaged in the regulation of negative emotions [43, 44]. Depressed individuals exhibit an exaggerated response to negative emotional stimuli and weakened cognitive control functions, which may be attributed to abnormal activity in the left MPFC [45]. Given the critical role of the left MPFC’s pivotal role in regulating positive emotions and inhibiting negative ones [46], we speculate that dysfunction in the left MPFC may contribute to the persistence of negative emotions, deficits in positive emotional experiences, and impaired cognitive control associated with depressive symptoms. When faced with complex tasks, as evidenced by fNIRS data obtained from the VFT, individuals with depressive symptoms may manifest flat affect, difficulties in language generation, and semantic retrieval. Consequently, alterations in fNIRS signals in the left MPFC could serve as a potential biomarker and predictor of depressive symptoms among Chinese university students.

However, this study has several limitations. First, the design of the VFT task requires rapid syllable changes every 20 seconds during the task period. This methodology was employed to reduce variability in silent intervals, thereby potentially diminishing activation effects associated with vocalization. Future research should consider incorporating more comprehensive cognitive paradigms to further elucidate the neural mechanisms underlying depressive symptoms. Additionally, fNIRS signals exhibit considerable inter-subject variability, attributable to differences in head size, head shape, and the spatial distribution of brain functional regions. Subsequent research will be conducted at the individual subject level, taking into account differences in head size and array placement. Second, multiple comparison correction methods were not utilized in the statistical analyses due to the small sample size. Feature selection aims to enhance the performance of prediction models and provide a deeper understanding of the data-generating process [47]. Previous research has shown that features can be selected as model inputs without correcting for multiple comparisons [48]. For instance, Fourdain *et al*. [49] presented the fNIRS results without applying Bonferroni or False Discovery Rate (FDR) correction. When the number of features exceeds the number of participants, this high feature-to-sample ratio may increase the risk of overfitting and reduce the generalizability of the classification results [50]. Future studies with larger sample sizes will facilitate the application of more rigorous multiple comparison correction methods. Third, the optimal KNN model did not achieve the desired accuracy. The PHQ-9 alone is not sufficiently rigorous for diagnosing depression. To fully realize its potential applicability, the classification model should be capable of accurately distinguishing the onset of depression and differentiating between mild, moderate, and severe forms of depression. Future research will explore the integration of deep learning models, multi-modal features, or the HAMD to potentially enhance classification accuracy. Lastly, our analysis did not include an external dataset for model validation and the absence of multicenter data from diverse regions may limit the representativeness of our findings. Without external validation, the model’s generalizability is potentially compromised. Moreover, training the model exclusively on participants from a single university introduces population homogeneity, further reducing representativeness. To establish the generalizability of the classification model among university students, future studies should attempt to validate the model using independent datasets.

## 5. Conclusion

Our fNIRS results demonstrate that Chinese university students with depressive symptoms exhibited significant alterations in frontotemporal activity. Among the five machine learning algorithms evaluated, the KNN model exhibited superior classification performance. The left MPFC contributed most to the KNN model’s classification accuracy, highlighting its central role in the emotional processing and cognitive flexibility impairments associated with depressive symptoms.

The integration of fNIRS with these machine learning classifiers holds potential for advancing the development of an objective, automated, and user-friendly assessment tool and intelligent system for detecting specific cerebral hemodynamic changes, thereby enhancing the early identification of depressive symptoms in Chinese university students. Future research should focus on validating these findings and refining classification models to improve their applicability on a large scale.

## Availability of Data and Materials

The fNIRS data in this study are available upon request. Further information and requests should be directed to and will be fulfilled by Dr. Yange Wei.
